# Nonlinear Heat Transport in Superlattices with Mobile Defects

**DOI:** 10.3390/e21121200

**Published:** 2019-12-06

**Authors:** David Jou, Liliana Restuccia

**Affiliations:** 1 Grup de Fisíca Estadística, Universitat Autònoma de Barcelona, 08193 Bellaterra, Catalonia, Spain; 2 Department of Mathematical and Computer Sciences, Physical Sciences and Earth Sciences, University of Messina, Viale F. Stagno d’Alcontres, Salita Sperone 31, 98166 Messina, Italy; lrestuccia@unime.it

**Keywords:** heat transport, extended thermodynamics, mobile defects, defect engineering, thermal transistor, superlattices

## Abstract

We consider heat conduction in a superlattice with mobile defects, which reduce the thermal conductivity of the material. If the defects may be dragged by the heat flux, and if they are stopped at the interfaces of the superlattice, it is seen that the effective thermal resistance of the layers will depend on the heat flux. Thus, the concentration dependence of the transport coefficients plus the mobility of the defects lead to a strongly nonlinear behavior of heat transport, which may be used in some cases as a basis for thermal transistors.

## 1. Introduction

The dependence of transport coefficients on the concentration of defects, applied stress, and nanostructure of the system allows new ways to achieve subtle and useful behaviors in energy management, thermal metamaterials, or thermal computation. uThis has given a strong impetus to defect engineering and nanoengineering to design and develop systems with suitable behaviors [1,2,3,4,5,6,7,8,9,10,11,12].

The aim of this paper is to analyze heat transport in superlattices with mobile point defects [13,14,15,16,17,18,19] as a thermodynamic exploration of possible metamaterials with sophisticated transport properties. The model provides a particular illustration of a much more general set of transport equations for anisotropic materials, and uses the fact that thermal conductivity may be strongly reduced by the presence of small amounts of point defects. If such defects may move inside the material under the influence of a heat flux, and if material barriers to the motion of defects may be provided by the interfaces of the superlattice, heat transport becomes a strongly nonlinear phenomenon. This may be used to control heat transfer in the superlattice by using the feedback of nonequilibrium distribution of defects on the value of the thermal resistance, and may be used in some cases as the basis for thermal transistors [20,21,22,23].

Though we use the formalism of classical irreversible thermodynamics, with fluxes being linear functions of the thermodynamic forces, the concentration dependence of the thermal conductivity establishes a deep coupling between the dynamics of defects and the heat transfer behavior, leading to globally nonlinear behavior. In Section 2, we present the model. In Section 3, we explore the heat-flux dependence of the thermal resistance of the layers constituting the superlattice; and in Section 4 we comment on possible applications of mobile defects as the basis for a thermal transistor. In Section 5, we consider coupled longitudinal and radial effects, for the sake of generality. Section 6 is devoted to conclusions and remarks.

## 2. The Model

**Figure 1:**
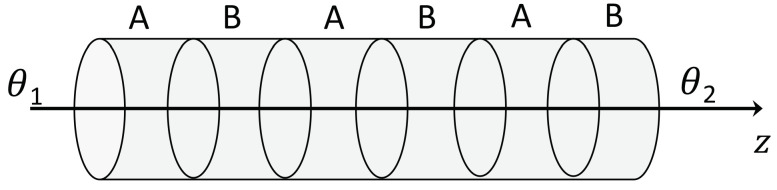
A superlattice of alternating layers of two semiconductors *A* and *B*.

We consider an elongated superlattice composed of alternating thin layers of materials A and B, see Figure 1.

Other geometries could be considered, but we take the simplest one allowing for an anisotropic system characterized by a longitudinal direction and two transversal directions with definitely different properties.

In each layer of material (or in some specific layers of material), there is some concentration *c* of point defects. In the absence of heat flux, this concentration is supposed to be homogeneous inside each layer. Consecutive layers are separated by a material interface which puts some barriers to heat flow and to flow of defects. The presence of these defects is not a consequence of a deficient fabrication method, but it is artificially controlled in order to modify in suitable ways the thermal conductivity of the material in each layer.

Indeed, it is known that the thermal conductivity of a material may be much reduced by the presence of a small amount of defects. This is the basis of the so-called “defect engineering of materials” and it is a recent field of research with a number of potential applications, such as in thermal metamaterials, heat diodes and heat transistors, improved photovoltaic devices or light-emitting devices, and so on [1,2,3,4,5,6,7,8,9,10,11,12].

Here, we will assume that such defects may move inside the material when the material is imposed with nonequilibrium boundary conditions [13,14,15,16,17,18,19]. In particular, we assume that they may move under the action of a heat flux—as the consequence, for instance, of some “phonon drag” phenomenon, or under an electric field, if the defects are charged.

The balance laws for specific internal energy *u* and defect concentration *c* are

$$c_{T}\dot{\theta }=-\nabla \cdot q,$$

(1) $$\dot{c}=-\nabla \cdot J,$$

where θ is the temperature, $c_{T}$ is the specific heat per unit volume (such that $du=c_{T}d\theta$), and q and J are the heat flux and the defect flow, respectively.

The classical entropy production per unit volume and time in a system with heat transport and defect transport is [24,25,26,27,28,29]

(2) $$\sigma =q\cdot \nabla \theta^{-1}-J\cdot \nabla \left(\mu \theta^{-1}\right),$$

with μ being the chemical potential of defects. This corresponds to the product of the fluxes of *u* and *c* times the gradients of the thermodynamic conjugates to *u* and *c*, namely, $\theta^{-1}$ and $-\mu \theta^{-1}$. Following classical irreversible thermodynamics, we will assume that the fluxes q and J may be expressed as linear combinations of the thermodynamic forces $\nabla \theta^{-1}$ and $-\nabla (\mu \theta^{-1})$.

**Figure 2:**
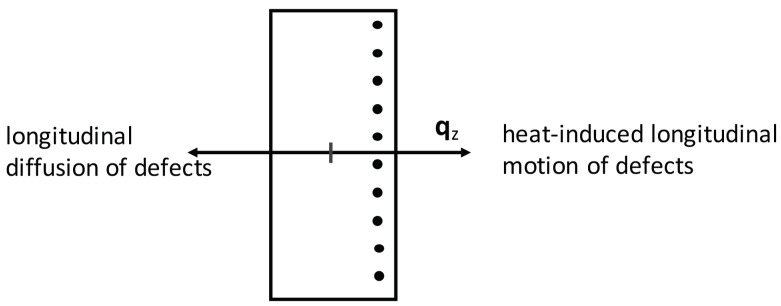
Heat-induced longitudinal motion of defects along the heat flux and longitudinal diffusion of defects against the gradient of defects concentration.

This may be written explicitly for longitudinal and radial components. Regarding the longitudinal components along the *z*-axis, we have (see Figure 2)

(3) $$\left(\begin{array}{c} q_{z}\\ J_{z} \end{array}\right)=\left(\begin{array}{cc} L_{qq} & L_{qc}\\ L_{cq} & L_{cc} \end{array}\right)\left(\begin{array}{c} \nabla_{z}\theta^{-1}\\ -\nabla_{z}\mu \theta^{-1} \end{array}\right),$$

with $L_{qq},L_{qc},L_{cq}$, and $L_{cc}$ being transport coefficients. The Onsager reciprocity relations state that the matrix of these coefficients must be symmetrical, i.e.,

(4) $$L_{qc}=L_{cq}.$$

Note that for some anisotropic systems a coupling between longitudinal and radial components is possible and it will be examined in Section 5, but here we stick to the simplest case.

Now, we will rewrite (3) in terms of ∇θ and of ∇c, by assuming that $\mu \theta^{-1}=\tilde{f}\left(c\right),$ so that $\nabla \left(\mu \theta^{-1}\right)=\frac{\partial \tilde{f}\left(c\right)}{\partial c}\nabla c=f_{\left(c\right)}\nabla c,$ with $\frac{\partial \tilde{f}\left(c\right)}{\partial c}=f_{\left(c\right)}.$ Then, (3) becomes

$$q_{z}=-\lambda \nabla_{z}\theta -\lambda^{\prime }\nabla_{z}c,$$

(5) $$J_{z}=-D^{\prime }\nabla_{z}\theta -D\nabla_{z}c,$$

where λ is the thermal conductivity, *D* is the diffusion coefficient of the defects, and $\lambda^{\prime }$ and $D^{\prime }$ are the coupling coefficients between defect field and heat flow field. Comparing (3) and (5), it is seen that $\lambda =L_{qq}\theta^{-2},\;D=L_{cc}f_{\left(c\right)},\;\lambda^{\prime }=L_{qc}f_{\left(c\right)}$ and $D^{\prime }=L_{cq}\theta^{-2}$.

Now, we assume that λ depends on *T* and on *c*. Thus, the dynamics of *c* will have an influence on the thermal conductivity. To explore this feature we rewrite (5) as

$$q=-\lambda^{{}^{\prime \prime }}\nabla \theta +\alpha_{1}cJ,$$

(6) $$J=-D^{{}^{\prime \prime }}\nabla c+\alpha_{2}cq,$$

where $\alpha_{2}cq$ is the drift velocity of defects under the action of a steady heat flux, $\lambda^{{}^{\prime \prime }}\equiv \lambda -\frac{\lambda^{\prime }D^{\prime }}{D}$, $\;\;\alpha_{1}c\equiv \frac{\lambda^{\prime }}{D}$, $\;\;D^{{}^{\prime \prime }}\equiv D-\frac{\lambda^{\prime }D^{\prime }}{D}$, $\;\;\alpha_{2}c\equiv \frac{D^{\prime }}{\lambda }$, and $\alpha_{1}cJ$ is related to the phonon flow induced by the motion of defects.

These transport equations apply to each layer of the superlattice, but transport through the interfaces between layers of materials *A* and *B* must be described by their own laws specifying the interface [1]. The equations are

(7) $$\Delta \theta =\theta_{A}-\theta_{B}\equiv R_{\theta AB}q_{z}+R_{\theta dAB}J_{z},$$

(8) $$\Delta c=c_{A}-c_{B}\equiv R_{dAB}J_{z}+R_{d\theta AB}q_{z}.$$

Here, $R_{\theta AB}$ and $R_{dAB}$ are respectively the thermal boundary resistance of the wall and the resistance of the wall to the flow of defects through it [1,2]. We will assume for simplicity that the coefficients of the crossed coupling terms are zero, namely, $R_{\theta dAB}=R_{d\theta AB}=0.$ Later on, we will focus the attention on some particular expression for these transport coefficients. In summary, since $\lambda^{\prime \prime }\left(\theta ,c\right)$ is a function of θ and *c*, the local motion of defects modifies the spatial distribution of *c* and therefore the value of $\lambda^{\prime \prime }$.

## 3. Longitudinal Heat Transport Across a Superlattice

We assume that the interfaces allow heat to pass, but that they do not allow the flow of defects from one layer to the neighboring layers, namely, that $R_{dAB}$ in (8) is very high. This assumption is for the sake of simplicity, as it allows one to consider the motion of defects as restricted to each particular layer. Since here we are only aiming to examine the general ideas of the model, rather than obtaining accurate particular realistic values, this approximation is sufficient for our purposes. We will consider that the whole system is submitted to a longitudinal heat flux as a consequence of a temperature difference along the long axis.

### 3.1. Steady State Distribution of Defects

After imposing $q_{z},$ we obtain from the second equation in (6) the distribution of defects in each layer of the material, in order to later consider the feedback of this concentration on the thermal resistance of each layer. Here, we consider the particularly simple but interesting case in which $D^{{}^{\prime \prime }}$ and $\alpha_{2}$ in (6) are constant. In a steady state, with J=0, the spatial distribution of defects inside a layer corresponding to $\dot{c}=0$ will be, by solving the second equation of (6),

(9) $$c\left(z\right)=c\left(0\right)exp\left[\frac{\alpha_{2}q}{D^{{}^{\prime \prime }}}z\right],$$

with c(0) related to the initial homogeneous concentration $c_{0}$ of defects in the layer at equilibrium. The value of c(0) may be related to $c_{0}$ by the conservation of the number of defects, according to (6) (namely, for J=0),

(10) $$c_{0}\;L=\int_{0}^{L}c\left(z\right)dz,\;c_{0}\;L=c\left(0\right)\int_{0}^{L}exp\left[\frac{\alpha_{2}}{D^{{}^{\prime \prime }}}qz\right]dz=\frac{c\left(0\right)D^{{}^{\prime \prime }}}{\alpha_{2}q}\left[exp\left(\frac{\alpha_{2}q}{D^{{}^{\prime \prime }}}L\right)-1\right].$$

Up to first order in $\frac{\alpha_{2}qL}{D^{{}^{\prime \prime }}}$, one has

(11) $$c\left(0\right)=\frac{c_{0}}{1+\frac{1}{2}\frac{\alpha_{2}qL}{D^{{}^{\prime \prime }}}}.$$

In the next Section, we consider how (10) and (11) modify the thermal resistence of the layer.

### 3.2. Nonlinear Thermal Resistance of the System

The thermal conductivity λ(θ,c) depends on the concentration of defects and on their spatial distribution in the system. We have seen in the previous Section that a longitudinal heat flux modifies the longitudinal distribution of mobile defects according to (9). Here, we consider how this modifies the effective thermal resistance (ThRes) of the corresponding layer. The thermal resistance of each layer is defined as

(12) $$q\equiv \frac{\theta \left(z_{1}\right)-\theta \left(z_{2}\right)}{ThRes},$$

with $z_{1}$ and $z_{2}$ being the positions of the boundaries of the layer. Note that this is analogous to Ohm’s law of electricity if *q* is replaced by the electrical current intensity and θ by the electrical potential. In our case, in Section 4, we have taken $z_{1}=0$, $z_{2}=L$.

### 3.3. Thermal Resistance as a Function of q

From Equation (6), for *q*, with J=0 (because in the steady state the total flow of defects is zero, since they are stopped by the interface) we have

(13) $$\theta \left(z_{1}\right)-\theta \left(z_{2}\right)=\int_{z_{1}}^{z_{2}}\frac{qdz}{\lambda^{{}^{\prime \prime }}\left(\theta (z),c(z)\right)},$$

where in the one-dimensional steady state, *q* is constant.

For the dependence of λ(θ,c) we assume

(14) $$\lambda \left(\theta ,c\right)=\frac{\lambda_{0}\left(\theta \right)}{1+a(\theta )c},$$

with a(θ) being a coefficient which depends on the nature of the material matrix and of the defects. This is a simple way of describing the reduction of thermal conductivity for increasing values of the concentration of defects. Since (14) may be written as

(15) $$\frac{1}{\lambda (\theta )}=\frac{1}{\lambda_{0}\left(\theta \right)}+\frac{a(\theta )c}{\lambda_{0}\left(\theta \right)},$$

and since λ is proportional to the collision time of the carriers, (15) may be interpreted as the fact that the total frequency of collisions of heat carriers $\frac{1}{\tau_{tot}}$ (with $\tau_{tot}$ being the average collision time of heat carriers in the presence of defects) is the sum of the frequency of collisions without defects, $\frac{1}{\tau_{0}}$, plus the frequency of collisions of the heat carriers with defects, assumed to be proportional to the defect concentration *c*. The simple model (14) as (15) could be improved at high concentration defects, but here it is not necessary for our illustrative purposes.

Ignoring the dependence of $\lambda_{0}\left(\theta \right)$ and a(θ) on θ (which could be easily implemented in a numerical model, but which is not necessary to get a qualitative understanding of the problem we are dealing with), we have

(16) $$\theta \left(z_{1}\right)-\theta \left(z_{2}\right)=\int_{z_{1}}^{z_{2}}\frac{q\left[1+ac(z)\right]dz}{\left(\lambda_{0}-\frac{\lambda^{\prime }D^{\prime }}{D}\right)-\frac{\lambda^{\prime }D^{\prime }}{D}ac\left(z\right)},$$

with c(z) given by (9). Since c(z) depends on *q*, (16) will lead to a thermal resistance depending on *q*. Indeed, (16) will be

(17) $$\theta \left(z_{1}\right)-\theta \left(z_{2}\right)=\frac{q}{\left(\lambda_{0}-\frac{\lambda^{\prime }D^{\prime }}{D}\right)}\int_{z_{1}}^{z_{2}}\frac{1+ac(z)dz}{1-Aac(z)},$$

with $A\equiv \left(\frac{\lambda^{\prime }D^{\prime }}{D}\right){\left[\lambda_{0}-\frac{\lambda^{\prime }D^{\prime }}{D}\right]}^{-1}$. This yields

(18) $$\theta \left(z_{1}\right)-\theta \left(z_{2}\right)=\frac{qL}{\lambda_{0}-\frac{\lambda^{\prime }D^{\prime }}{D}}\left\{1+\frac{D(1+A)}{\alpha_{2}qAL}ln\left[\frac{1-Aac\left(0\right)exp\left[\frac{\alpha_{2}q}{D^{{}^{\prime \prime }}}L\right]}{1-Aac(0)}\right]\right\},$$

with *q* being the modulus of the heat flux q.

Up to the first order in $\frac{ac\left(0\right)q\alpha_{2}L}{D^{{}^{\prime \prime }}}$ and for $z_{1}=0,\;z_{2}=L$, one has

(19) $$\theta \left(0\right)-\theta \left(L\right)=\frac{qL}{\lambda_{0}-\frac{\lambda^{\prime }D^{\prime }}{D}}\frac{1}{1-Aac(0)}\left\{1+\frac{ac\left(0\right)\alpha_{2}qL}{2D^{{}^{\prime \prime }}}\left[\frac{1}{1-Aac(0)}\right]\right\}.$$

The thermal resistance will then have the form

(20) $$TherRes\left(\theta ,c,q\right)=TherRes\left(\theta ,c\right)\left\{1+ac\left(0\right)\left[\frac{\alpha_{2}L}{2D^{\prime \prime }}(1-Aac\left(0\right)\right]q\right\},$$

with TherRes(θ,c) being the part of the thermal resistance which does not depend on the heat flux, i.e., which does not depend on the mobility of defects (recall that the heat-induced motion of defects is described by the coefficient $\alpha_{2}$, when this coefficient is zero the defects do not move). Then, the thermal resistance depends on *q* and, in the present approximation, it increases with *q* and with c(0). A possible application of this dependence could be in situations requiring some stability of the value of the heat flux in front of changes of temperature in one of the boundaries of the system. Indeed, the heat-flux dependence of (19) will make that an increase in θ(0) (at constant θ(L)) will produce an increase of the heat flux lower than the increase corresponding to a thermal resistance independent on *q* (namely, lower than for $\alpha_{2}=0$ in (20)).

The total thermal resistance of a superlattice is the sum of the thermal resistances of the layers plus those of the interfaces. To these effects, some phonon coherence effects may also arise, related to the thickness of the layers [30,31]. In the situations we are considering, the wave nature of phonons is not expected to be relevant, because the defects will make the phonon flow incoherent. Thus, the total thermal resistance will be the sum of thermal resistance of the layers (related to the thickness of the layers) and those of the interfaces (related to the physical differences between the layers in contact).

In Equation (20), we have expressed how the mobility of defects influences the thermal resistance of a layer. Different layers could have different concentrations of defects (different values of c(0)) and different kinds of defects (different defect mobilities $\alpha_{2}$). The thermal resistance of the interfaces between layers would also be modified by the heat flux, as a consequence of the defect mobility. We have considered that the interfaces do not allow the flow of defects across them and that, as a consequence, defects will accumulate in one side of the interface (that to which the defects are arriving as a consequence of being dragged by the heat flux) and will be depleted from the other side of the interface (that from which the defects are leaving because of being dragged). In general, the thermal resistance of the interfaces is higher when the physical differences of nature, structure, and composition of the layers in contact are higher. Thus, since the drag of defects increases the difference of defect concentrations at both sides of the interface (increasing it at one side and decreasing it at the opposite side), the thermal resistance of the interfaces will increase with the heat flux. Concrete expressions for this increase will depend on the model adopted for heat transfer across the interface [32,33].

## 4. Transversal Heat Transport: A Mathematical Model for a Defect-Based Thermal Transistor

Thermal transistors play, with respect to the heat flux, an analogous role to electronic transistors with respect to electric currents, namely, they may control and amplify a heat flux [20,21,22,23].

Currently, they may be useful for the control of heat flux in small scale devices. In the future, they could be the basis of logical gates and of thermal computers processing information in form of thermal signals. Several different strategies are being proposed to obtain heat transistors, namely, thermoelastic, electrochemical, thermoelectrical, and quantum—[34,35,36,37,38], respectively. In this Section, we propose a further new strategy based on the heat-dependence of thermal resistance, that we have outlined in the previous Section, but used in a transversal way, rather than in a longitudinal way.

**Figure 3:**
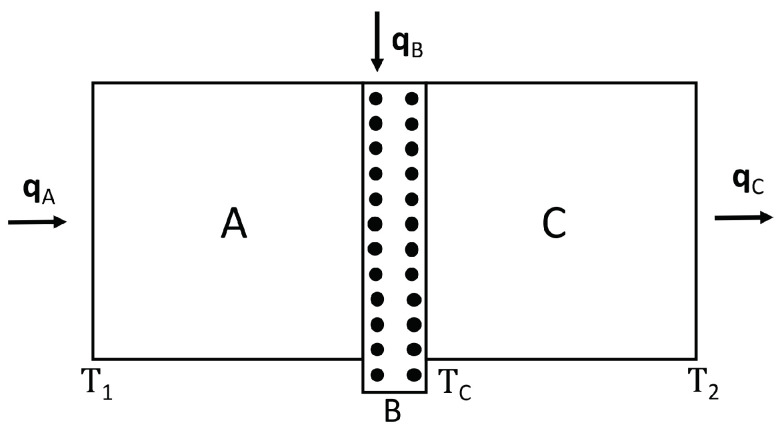
Heat transistor system, based on mobile defects driven by a heat flux considered in this Section.

The system is sketched in Figure 3.

In our proposal, a thin layer of a material B containing mobile defects able to move along the layer, is sandwiched between two pieces of materials A and C. Part A is traversed by a heat flux $q_{A}$, perpendicular to the B thin layer, and along the layer B a heat flux $q_{B}$ is injected. The total heat supplied to A and B per unit of time flows out of the system through part C. The heat flux $q_{B}$ produces a drift of defects along the direction $q_{B}$, thus, the heat flux *B* carries out a number of defects from region *B* and, as a consequence, increases the thermal conductivity of layer *B*. To make easier the removal of such defects while $q_{B}$ is flowing, we make layer B a little bit longer (in the direction of $q_{B}$) than the width of sections A and C. Note that, in contrast to Section 3, in the present Section, the defects are dragged in a direction transversal to the longitudinal axis of the superlattice.

In order for this system to be considered as a transistor, it is necessary that

(21) $$\frac{\partial q_{C}}{\partial q_{B}}>1.$$

This implies that the variations in the outgoing heat flow $q_{C}$ are amplified through variations of $q_{B}$.

The equations describing the fluxes $q_{A}$ and $q_{C}$ between the positions characterized by temperatures $T_{1}$ and $T_{C}$, and between $T_{C}$ and $T_{2}$, respectively, (see Figure 3) are

(22) $$T_{1}-T_{C}=\left[\frac{L_{A}}{\lambda_{A}}+\frac{L_{B}}{\lambda_{B}\left(q_{B}\right)}\right]q_{A}=R_{AB}\left(q_{B}\right)q_{A},$$

(23) $$T_{C}-T_{2}=\frac{L_{C}}{\lambda_{C}}q_{C}=R_{C}q_{C},$$

with $R_{AB}$ and $R_{C}$ being the thermal resistances of A+B and of *C*, respectively. In this simple formulation, we neglect the thermal resistances at the interfaces AB and BC, but there is not difficulty in incorporating them in a more accurate but more cumbersome analysis. Equations (22) and (23) follow from direct application of Fourier’s law. The new point is that $R_{AB}$ depends on the flux $q_{B}$.

The value of $T_{C}$ is found from the steady-state condition $q_{C}=q_{A}+q_{B}$. This implies that

(24) $$R_{C}^{-1}\left[T_{C}-T_{2}\right]=R_{AB}^{-1}\left[T_{1}-T_{C}\right]+q_{B}.$$

From here, for $T_{C}$ as a function of $q_{B}$, one obtains

(25) $$T_{C}=\frac{R_{AB}^{-1}T_{1}+R_{C}^{-1}T_{2}+q_{B}}{R_{AB}^{-1}+R_{C}^{-1}}.$$

Introducing this expression for $T_{C}$ into Equation (22), one obtains

(26) $$q_{A}=R_{AB}^{-1}T_{1}-\frac{R_{AB}^{-1}}{R_{AB}^{-1}+R_{C}^{-1}}\left[R_{AB}^{-1}T_{1}+R_{C}^{-1}T_{2}+q_{B}\right].$$

From here, the relation $\frac{\partial q_{A}}{\partial q_{B}}$ may be obtained, taking into account that $R_{AB}^{-1}$ depends on $q_{B}$, since an increase in $q_{B}$ produces a decrease of $R_{AB}$. From here, we obtain the amplification factor

$$\frac{\partial q_{C}}{\partial q_{B}}=1+\frac{\partial q_{A}}{\partial q_{B}}=1-\frac{R_{AB}^{-1}}{R_{AB}^{-1}+R_{C}^{-1}}\left[1+\Gamma_{AB}T_{1}\right]+$$

(27) $$+\Gamma_{AB}T_{1}-\frac{R_{AB}^{-1}T_{1}+R_{C}^{-1}T_{2}+q_{B}}{{\left[R_{AB}^{-1}+R_{C}^{-1}\right]}^{2}}T_{AB}R_{C}^{-1},$$

with $\Gamma_{AB}$ standing for $\Gamma_{AB}\equiv \frac{\partial R_{AB}^{-1}}{\partial q_{B}}$. If $\Gamma_{AB}=0$, (27) reduces to

(28) $$\frac{\partial q_{C}}{\partial q_{B}}=\frac{R_{C}^{-1}}{R_{AB}^{-1}+R_{C}^{-1}}<1.$$

In our case, since $R_{AB}=R_{A}+R_{B}\left(q_{B}\right)$, and $R_{B}$ (namely, $\frac{L_{B}}{\lambda_{B}\left(q_{B}\right)}$) decreases with an increase of $q_{B}$, one has $\frac{\partial R_{AB}}{\partial q_{B}}<0$, and thus, $\Gamma_{AB}=-R_{AB}^{-2}\frac{\partial R_{AB}}{\partial q_{B}}>0$. From (27), it follows that the amplification factor will be higher than 1 if

(29) $$\Gamma_{AB}>\frac{R_{AB}^{-1}}{R_{C}^{-1}}+\frac{1}{T_{1}-T_{2}}.$$

In order to modelize how $q_{B}$ reduces the total concentration of defects in the layer *B*, assume that the flux of defects is

(30) $$J_{def}=-D\nabla c+c\alpha q,$$

with αq giving the drift velocity of defects under the presence of a heat flow.

Thus, in steady state, we have

(31) $$c\left(z\right)=c\left(0\right)exp\left[\frac{\alpha q}{D}z\right].$$

For q=0, the concentration of defects is homogeneous in the layer *B*. The higher the *q*, the shorter the characteristic length l≡D/αq, where the defects become concentrated. In our model, we propose that the layer B has a length wider than the width of A and C. In this way, a fraction of defects will accumulate in this extra zone, and will go out from the region where they reduce the heat flux. The effective concentration of defects in the zone of the heat flow will be reduced, and the reduction of thermal resistance for a given heat flux will be more effective the longer is the additional length *d* of the layer.

For the sake of a simple illustration, assume that

(32) $$\lambda_{B}\left(T,c\right)=\frac{\lambda_{B0}\left(T\right)}{1+\beta T_{C}}\approx \lambda_{B0}\left(T\right)\left[1-\beta T_{C}\right],$$

as it was been assumed in (14).

We will have

(33) $$\frac{\partial R_{B}}{\partial q}=\frac{L_{B}}{\lambda_{B0}}\beta T\frac{\partial c}{\partial q}<0,$$

i.e.,

(34) $$\Gamma_{AB}=-\frac{L_{B}}{\lambda_{B0}R_{AB}^{2}}\beta T\frac{\partial c}{\partial q}.$$

In view of relation (29), this means that the present model will work as a thermal transistor provided that

(35) $$-\frac{\partial c}{\partial q}=\frac{\lambda_{B0}}{L_{B}}\frac{R_{AB}R_{C}}{\beta T_{c}\left(T_{1}-T_{c}\right)}.$$

To have this behavior, it will be convenient that α is high, *D* is low, and the additional depth *d* of layer *B* is relatively long.

## 5. General Case with Longitudinal and Transversal Components of **q** and **J**

**Figure 4:**
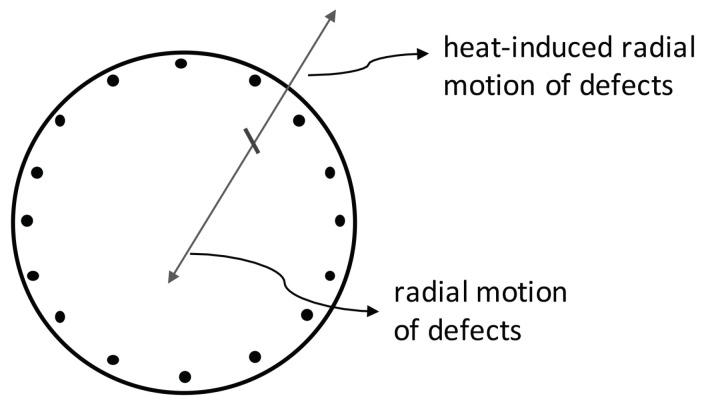
Heat-induced radial motion of defects and radial diffusion of defects.

In this Section, we will consider the additional possibility that a longitudinal heat flow produces not only a longitudinal drag of defects but also a transversal drag of defects. This is possible in some anisotropic materials. From the entropy production (2), in classical nonequilibrium thermodynamics, the equations relating the fluxes q, J to their thermodynamic forces $\nabla \theta^{-1}$ and $-\nabla (\mu \theta^{-1})$, in the case where we consider the longitudinal and transversal components of these fields, see Figure 1 and Figure 4, we have

(36) $$\left(\begin{array}{c} q_{z}\\ q_{r}\\ J_{z}\\ J_{r} \end{array}\right)=\left(\begin{array}{cccc} \lambda_{zz} & \lambda_{zr} & \lambda_{zz}^{\prime } & \lambda_{zr}^{\prime }\\ \lambda_{rz} & \lambda_{rr} & \lambda_{rz}^{\prime } & \lambda_{rr}^{\prime }\\ \chi_{zz}^{\prime } & \chi_{zr}^{\prime } & \chi_{zz} & \chi_{zr}\\ \chi_{rz}^{\prime } & \chi_{rr}^{\prime } & \chi_{rz} & \chi_{rr} \end{array}\right)\left(\begin{array}{c} \nabla_{z}\theta^{-1}\\ \nabla_{r}\theta^{-1}\\ -\nabla_{z}\mu \theta^{-1}\\ -\nabla_{r}\mu \theta^{-1} \end{array}\right)$$

The Onsager reciprocity relations state that the matrix of these coefficients must be symmetrical, i.e., we have

(37) $$\lambda_{zr}=\lambda_{rz},\;\lambda_{zz}^{\prime }=\chi_{zz}^{\prime },\;\lambda_{zr}^{\prime }=\chi_{rz}^{\prime },\;\lambda_{rz}^{\prime }=\chi_{zr}^{\prime },\;\chi_{zr}=\chi_{rz},\;\lambda_{rr}^{\prime }=\chi_{rr},\;\chi_{rz}=\chi_{zr}.$$

Note that for some anisotropic systems, a coupling between longitudinal and radial components is possible. When radial effects are neglected, (36) reduces to (3).

Now, we will rewrite (36) in terms of ∇θ and of ∇c, by assuming that $\mu \theta^{-1}=\tilde{f}\left(c\right),$ so that $\nabla \left(\mu \theta^{-1}\right)=\frac{\partial \tilde{f}\left(c\right)}{\partial c}\nabla c=f_{\left(c\right)}\nabla c,$ with $\frac{\partial \tilde{f}\left(c\right)}{\partial c}=f_{\left(c\right)}.$ Then, (3) becomes

$$q_{z}=-\lambda_{zz}\theta^{-2}\nabla_{z}\theta -\lambda_{zr}\theta^{-2}\nabla_{r}\theta -\lambda_{zz}^{\prime }f_{\left(c\right)}\nabla_{z}c-\lambda_{zr}^{\prime }f_{\left(c\right)}\nabla_{r}c,$$

$$q_{r}=-\lambda_{rz}\theta^{-2}\nabla_{z}\theta -\lambda_{rr}\theta^{-2}\nabla_{r}\theta -\lambda_{rz}^{\prime }f_{\left(c\right)}\nabla_{z}c-\lambda_{rr}^{\prime }f_{\left(c\right)}\nabla_{r}c,$$

$$J_{z}=-\chi_{zz}^{\prime }\theta^{-2}\nabla_{z}\theta -\chi_{zr}^{\prime }\theta^{-2}\nabla_{r}\theta -\chi_{zz}f_{\left(c\right)}\nabla_{z}c-\chi_{zr}f_{\left(c\right)}\nabla_{r}c,$$

(38) $$J_{r}=-\chi_{rz}^{\prime }\theta^{-2}\nabla_{z}\theta -\chi_{rr}^{\prime }\theta^{-2}\nabla_{r}\theta -\chi_{rz}f_{\left(c\right)}\nabla_{z}c-\chi_{rr}f_{\left(c\right)}\nabla_{r}c.$$

In a more compact version, we could write (38) in a form analogous to (6) but with matricial form of λ, D, $\alpha_{1}$ and $\alpha_{2}$, namely,

$$q=-\lambda \cdot \nabla \theta +\alpha_{1}c\cdot J$$

(39) $$J=-D\cdot \nabla c+\alpha_{2}c\cdot q,$$

with D being the diffusion coefficient of defects and $\alpha_{2}q$ giving the drift velocity of defects under the action of a steady heat flux.

These transport equations apply to each layer of the superlattice, but transport through the interfaces between layers must be described by their own laws specifying the interface. The equations are like (7) and (8), namely,

(40) $$\Delta \theta =\theta_{A}-\theta_{B}\equiv R_{\theta AB}q_{z}+R_{\theta dAB}J_{z},$$

(41) $$\Delta c=c_{A}-c_{B}\equiv R_{dAB}J_{z}+R_{d\theta AB}q_{z}.$$

Here, $R_{\theta AB}$ and $R_{dAB}$ are respectively the thermal boundary resistance of the wall and the resistance of the wall to the flow of defects through it. We will assume for simplicity that $R_{\theta dAB}=R_{d\theta AB}=0.$ Eventually, we considered that the transport coefficients depend on θ and c, i.e., we have $\lambda_{ij}\left(\theta ,c\right),\;\lambda_{ij}^{\prime }\left(\theta ,c\right),\;\chi_{ij}\left(\theta ,c\right),$$\;\chi_{ij}^{\prime }\left(\theta ,c\right).$ Later on, we will specify some expressions for these transport coefficients.

We will assume that $R_{dAB}$ is very high, i.e., that the interfaces do not allow the flow of defects from one layer to the neighboring layers. This assumption is for the sake of simplicity, as it allows one to consider the motion of defects as localized to each particular layer.

We will consider that the whole system is submitted to a temperature difference along the long axis, namely, it is submitted to a temperature gradient $\nabla_{z}\theta ,$ which will depend on the position along the axis. Instead, the longitudinal heat flux $q_{z}$ will be constant along the axis in the steady state. After imposing $q_{z},$ our aim is to obtain the distribution of defects in each layer of the material and the feedback of this concentration on the thermal resistance of each layer.

To have a maximum simplicity, we consider a $q_{z}$ imposed on the system and reduce the equations for q to

(42) $$q_{z}=-{\tilde{\lambda }}_{zz}\left(c\right)\nabla_{z}\theta ,$$

i.e., we assume that the radial gradient of θ is negligible with respect to its longitudinal gradient along *z*. In a steady state, the spatial distribution of defects inside a layer corresponding to $\dot{c}=0$ will be

(43) $$c\left(z\right)=c\left(0\right)exp\left[\frac{\alpha_{z}q}{D}z\right].$$

We consider in more detail the equations for the defects as

(44) $$J_{r}=-{\tilde{\chi }}_{rz}\nabla_{z}\theta -{\tilde{\chi^{\prime }}}_{rz}\nabla_{z}c-{\tilde{\chi }}_{rr}\nabla_{r}c,$$

(45) $$J_{z}=-{\tilde{\chi }}_{zz}\nabla_{z}\theta -{\tilde{\chi^{\prime }}}_{zz}\nabla_{z}c-{\tilde{\chi }}_{zr}\nabla_{r}c,$$

where we assume that the radial gradient of concentrations is not necessarily negligible. The first terms on the right hand side of Equations (44) and (45) describe the motion of point defects produced by the heat flux, and the other two terms describe diffusion of defects in longitudinal and radial directions.

In terms of $q_{z}$ (i.e., expressing $\nabla_{z}\theta$ in terms of $q_{z}$) and in the steady state ($J_{r}=0,J_{z}=0$), Equations (44) and (45) may be rewritten as

(46) $$\frac{{\tilde{\chi }}_{rz}}{{\tilde{\lambda }}_{zz}}q_{z}=-D_{rz}\nabla_{z}c-D_{rr}\nabla_{r}c,$$

(47) $$\frac{{\tilde{\chi }}_{zz}}{{\tilde{\lambda }}_{zz}}q_{z}=-D_{zz}\nabla_{z}c-D_{zr}\nabla_{r}c,$$

with $D_{ij}$ being the components of the tensorial diffusion coefficient of point defects. From Equations (46) and (47), the spatial distribution of defects c(r,z) in the steady state under the presence of heat flux $q_{z}$ may be obtained. In fact, Equations (46) and (47) describe the transversal and longitudinal effect of heat flux on the point defects which are sketched in Figure 1 and Figure 2. Thus, the defects will also flow towards the lateral walls of the superlattice. Then, two effects will be competing in the modification of thermal resistance in terms of the heat flux: an increase due to longitudinal accumulation; and a decrease due to a radial accumulation near the walls. The examination of this situation is much more complex than in Section 3.

## 6. Concluding Remarks

In this paper, we have worked out a simple transport equation to describe heat transfer in systems with mobile defects. The heat flux modifies the spatial distribution of defects, and the defects modify the thermal resistance of the layers and the interfaces, thus, influencing the heat flux itself. This is also found, for instance, in heat transport in turbulent superfluid helium, where the heat flux produces quantized vortices which contribute to the thermal resistance of the system [39]. In particular, we have worked out a simplified model of how the effective thermal resistance of a layer of a thermal superlattice may depend on q as a result of q inducing a motion of point defects and that the defects are stopped at the interfaces. The effects found here could contribute to a relative stabilization of the heat flux, by reducing the variation of q following from a variation of the boundary temperature. Note that since $q_{z}>0$ as $q_{z}<0$ produce different nonequilibrium spatial distributions of the defects, this will imply some heat rectification. Furthermore, we have considered a possible thermal transistor, in which a transversal heat flux controls the thermal resistance through a spatial redistribution of defects. This suggests a new way of achieving thermal transistors, besides the ways previously suggested in the literature.

The effects proposed here could be reinforced by including temperature dependence of the concentration-dependent contribution to thermal conductivity (second term of Equation (43)). If the contribution of *c* is multiplied by an increasing function of temperature, the dependence of the thermal resistance of the defect layer will increase in a stronger way with increasing heat flux.

It is also interesting to note that the different behavior of the interface with respect to heat flux and defect flux breaks the Onsager reciprocity at a macroscopic level, though it remains valid at a microscopic level. Indeed, in (23) we have assumed Onsager symmetry of the transport coefficients inside any layer of the superlattice. Thus, a temperature gradient contributes to a defect flux, and a concentration gradient contributes to a heat flux. However, since the interfaces allow a heat flux but not a defect flux through them, imposing a temperature gradient will not allow a defect flux in the steady state.
